# Intraoperative TOE guided management of newly diagnosed severe tricuspid regurgitation and pulmonary hypertension during orthotopic liver transplantation: a case report demonstrating the importance of reversibility as a favorable prognostic factor

**DOI:** 10.1186/s12871-019-0795-6

**Published:** 2019-07-13

**Authors:** B. Pearce, R. Hu, F. Desmond, D. Banyasz, R. Jones, C. O. Tan

**Affiliations:** 1 Department of Anaesthesia and Pain Medicine, Austin Health, 145 Studley Rd Heidelberg, Melbourne, Victoria 3084 Australia; 2 Honorary Clinical Lecturer, Anaesthesia, Perioperative and Pain Medicine Unit, Melbourne Medical School, The University of Melbourne, Parkville, Australia; 3 Department of Surgery, Melbourne Medical School, The University of Melbourne, Melbourne, Victoria Australia; 4 Liver Transplant Service, Austin Hospital, Austin Health, 145 Studley Rd, Heidelberg, Victoria 3084 Australia

**Keywords:** Liver transplantation, Tricuspid regurgitation, Pulmonary hypertension, Central venous hypertension, Pulmonary artery catheter, Trans-oesophageal echocardiography, case report

## Abstract

**Background:**

Tricuspid regurgitation (TR) and pulmonary hypertension (PHT) are highly dynamic cardiovascular lesions that may progress rapidly, particularly in the orthotopic liver transplantation (OLT) waitlist population. Severe TR and PHT are associated with poor outcomes in these patients, however it is rare for the two to be newly diagnosed intraoperatively at the time of OLT. Without preoperative information on pulmonary vascular and right heart function, the potential for reversibility of severe TR and PHT is unclear, making the decision to proceed to transplant fraught with difficulty.

**Case presentation:**

We present a case of successful orthotopic liver transplantation (OLT) in a 48 year old female with severe (PHT) (mean pulmonary arterial pressure > 55 mmHg) and severe TR diagnosed post induction of anaesthesia. The degree of TR was associated with systemic venous pressures of > 100 mmHg resulting in massive haemorrhage during surgery and difficulty in distinguishing venous from arterial placement of vascular access devices. Intraoperative transoesophageal echocardiography (TOE) proved crucial in diagnosing functional TR due to tricuspid annular and right ventricular (RV) dilatation, and dynamically monitoring response to treatment. In response to positioning, judicious volatile anaesthesia administration, pulmonary vasodilator therapy and permissive hypovolemia during surgery we noted substantial improvement of the TR and pulmonary arterial pressures, confirming the reversibility of the TR and associated PHT.

**Conclusion:**

TR and PHT are co-dependent, dynamic, load sensitive right heart conditions that are interdependent with chronic liver disease, and may progress rapidly in patients waitlisted for OLT. Use of intraoperative TOE and pulmonary artery catheterisation on the day of surgery will detect previously undiagnosed severe TR and PHT, enable rapid assessment of the cause and the potential for reversibility. These dynamic monitors permit real-time assessment of the response to interventions or events affecting right ventricular (RV) preload and afterload, providing critical information for prognosis and management. Furthermore, we suggest that TR and PHT should be specifically sought when waitlisted OLT patients present with hepatic decompensation.

**Electronic supplementary material:**

The online version of this article (10.1186/s12871-019-0795-6) contains supplementary material, which is available to authorized users.

## Background

Tricuspid regurgitation (TR) shares a complex relationship with chronic liver disease (CLD). Commonly seen in association with Pulmonary hypertension (PHT), TR severity more than mild is independently associated with cholestatic derangements in ‘liver function tests’ (LFTs), and the strength of the association dependent on TR severity [1]. The subsequent increase in central venous pressures reduces hepatic perfusion pressure, adding ischaemic stress to the liver and, increases venous bleeding during OLT. Liver disease itself may cause or worsen functional TR (i.e.TR without structural abnormality of the valve leaflets [2]) by increasing RV afterload or RV preload. This may be caused by an increased pulmonary vascular resistance due to porto-pulmonary hypertension (PoPH) or an increased pulmonary vascular blood volume due to a high cardiac output and/or fluid overload, often referred to as circulatory overload, or by impairing left ventricular function and increasing left atrial pressure (LAP) [3], due to cirrhotic cardiomyopathy. Other cause of pulmonary hypertension may also be present, such as pulmonary emboli, left ventricular failure and respiratory disease. The increase in RV preload also seen in liver disease worsens functional TR via an increase in systemic venous blood volume. Determining the cause is crucial for accurate prognostication and treatment. Severe PHT (defined as a mean PAP > 45 mmHg) associated with CLD is considered an absolute contraindication to transplantation in many centres worldwide [4]. Thus aggressive management of PHT in these patients allows more to proceed to transplant.

The highly load dependent nature and potential rapid progression of functional TR increases the likelihood of patients presenting for OLT with new onset severe TR. International liver transplant waitlist guidelines variably recommend transthoracic echocardiography (TTE) from every 3 months to 12 months; however evidence supporting the efficacy of this timing for detection of severe TR prior to OLT is lacking. Due to the co-dependent nature of TR, PHT and CLD, precipitants of hepatic decompensation may acutely worsen TR, and significant TR can worsen hepatic function, resulting in a rapid deterioration of both right heart function and liver function. Thus it is important to exclude right heart lesions when an acute deterioration of liver function is noted.

When TR and/or PHT are identified in an OLT waitlisted patient, determining the cause and subsequently optimizing cardiac function, volume state, and PHT is associated with reduced postoperative mortality [4, 5]. In particular, pulmonary vasodilator therapy, initiated pre-operatively, has been successfully used to lower PAP and to improve outcomes in some patients [4–6]. When diagnosed incidentally on the operating table however, opportunity for comprehensive assessment and establishment of reversibility is limited. We present a case of severe TR and PHT diagnosed after induction of anaesthesia for OLT, with successful management demonstrating reversibility of both TR and PHT and subsequent good postoperative outcome.

## Case presentation

Informed consent was obtained from the patient for the presentation of this case report with associated figures. A 48 year-old female patient, was scheduled for urgent OLT after an acute on chronic deterioration in hepatic function on a background of Caroli’s disease, non-alcoholic steato-hepatitis (NASH) and possible haemochromatosis. She had a known history of portal hypertension with oesophageal varices, ascites, and encephalopathy. Her past medical history also included morbid obesity and mild congenital cognitive impairment.

Echocardiography performed 18 months prior to transplantation demonstrated normal left and right ventricular (RV) systolic function and trivial TR. PAP was considered to be low but could not be estimated due to the minimal TR jet. Hence she did not receive a right heart catheterization study. At the time of waitlising her MELD (Model for End Stage Liver Disease) score was 16. However, her hepatic and renal function deteriorated in the six weeks prior to transplant, resulting in hospitalisation. This deterioration was triggered by an abdominal mesh infection. She was noted to have tender hepato-splenomegaly on admission. She was admitted to the intensive care unit (ICU) and placed on a terlipressin infusion twice during her admission; on both occasions however, her renal and hepatic function continued to deteriorate. Her surgery was therefore expedited. A repeat transthoracic echocardiogram was requested but not performed by the time of surgery; bedside TTE at time of surgery was considered but no trained staff were available to perform this test. Immediately prior to surgery her MELD score had risen to 39, with a bilirubin of 1062 micromol/L and a creatinine of 262 micromol/L.

**Fig. 1:**
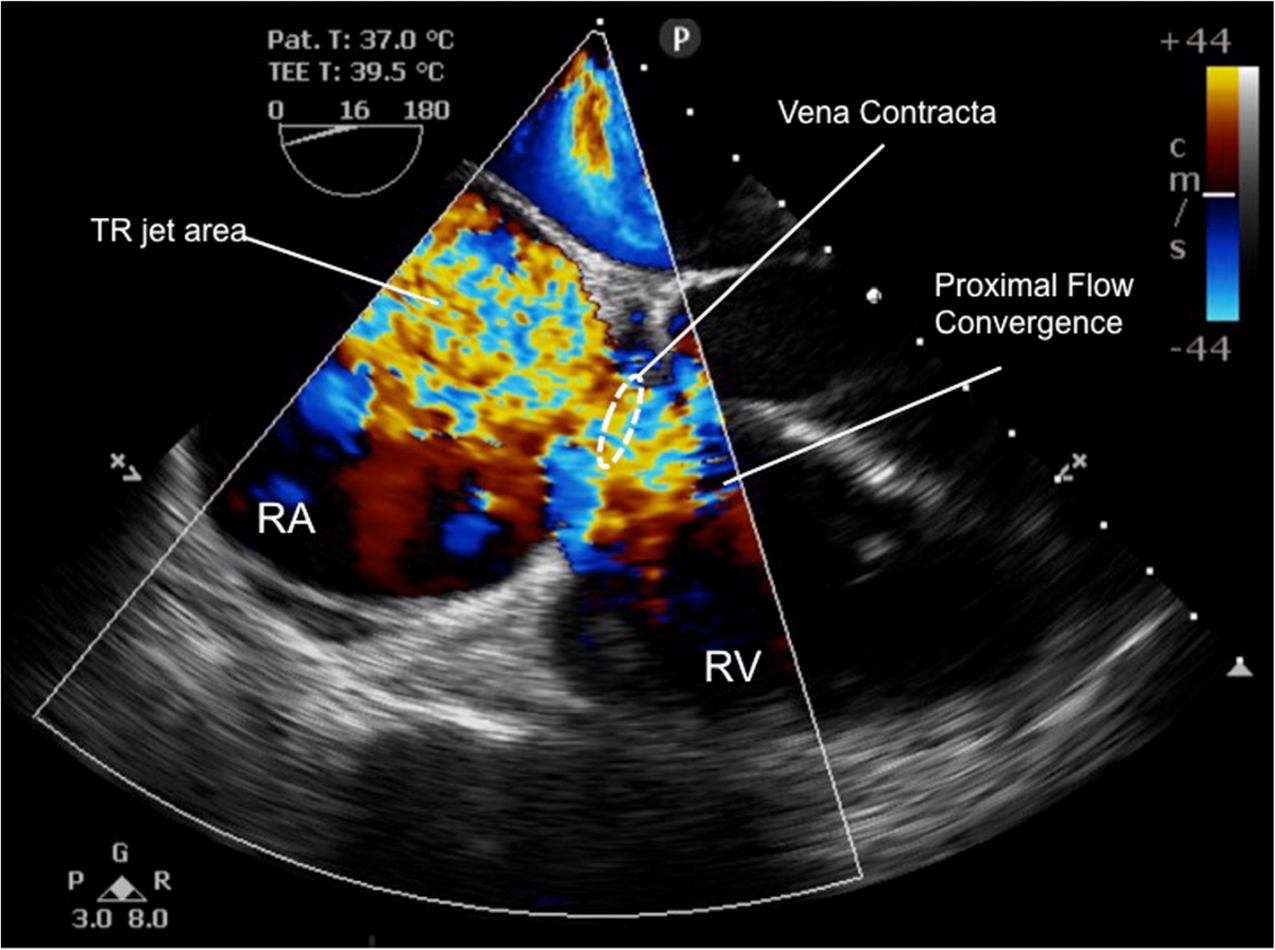
Severe tricuspid regurgitation, mid-oesophageal 4-chamber view at frame of maximal systolic TR jet area, after induction of anaesthesia


As per our instutional practice, anaesthesia was induced using a modified rapid sequence induction with propofol 100 mg, fentanyl 500mcg and suxamethonium 100 mg before proceeding with invasive monitoring. Anaesthesia was maintained with isoflurane to achieve an age-corrected mean alveolar concentration (MAC) of 0.8–1.0 together with fentanyl and cisatracrium infusions titrated to a bisprectral index (BIS™, Medtronic, Minneapolis, MN) of 40–60. The patient had a hyperdynamic circulation with radial arterial pressures of > 160 mmHg throughout induction with an end-tidal carbon dioxide (etCO_2_) of 42 mmHg. On needle cannulation of the right internal jugular vein under ultrasound guidance using a three-lumen central venous catheter kit (Arrow Medical, Kington, UK), pulsatile flow was observed. Blood gas analysis confirmed venous sampling (PO_2_ of 56 mmHg). Intravenous placement of the wire placement was confirmed on ultrasound. Transducing the central venous catheter demonstrated a peak pressure of 105 mmHg with evidence of pulsatile variation distinct from the radial arterial pressure trace. Subsequent cannulation for PAC insertion using a 9French sheath (Edwards Lifescience, Irvine, California) was again associated with high systemic venous pressures and a highly pulsatile venous waveform. Systolic pulmonary arterial pressure (sPAP) measured 90 mmHg and mean pulmonary artery pressure (mPAP) was 55 mmHg. The PAC could not be wedged and we were therefore unable to estimate LAP and measure pulmonary vascular resistance (PVR). The measured cardiac index (CI) was 2.5 by PAC thermodilution (CCombo pulmonary artery catheter, Edwards Lifesciences, Irvine, California). This was regarded as underestimating the true CI because of the severe TR. The decision was made at this point to use intraoperative TOE monitoring to assess valvular function, biventricular volume state and systolic function. TOE examination revealed normal LV and RV systolic function, but with severe TR (jet area 14cm^2^ [severe >10cm^2^]; vena contracta width 9.6 mm [severe > 7 mm]) and RV dilatation (RV end diastolic area 47cm^2^ [normal < 25 cm^2^]). (Fig. 1, Additional file 1: Movie Clip 1).

**Additional file 1**: Movie Clip 1 (MP4 16544 kb)


The findings of severe TR, grossly elevated systemic venous pressures, severe PHT, and dilated RV were inconsistent with her prior TTE examination findings. Her severely elevated CVP and mPAP of > 45 mmHg raised serious concerns regarding hepatic perfusion pressure into the newly grafted liver, potentially uncontrollable venous bleeding intraoperatively, and the likelihood of a poor postoperative outcome after transplantation. With her SBP trending at 160 mmHg we considered inadequate anaesthesia as a potentially confounding factor to her PHT despite acceptable BIS values and thus went on to administer further isoflurane anaesthesia. Several interventions were instituted to assess the reversibility of the patient’s TR and PHT; reverse Trendellenberg positioning, hyperventilation to etCO_2_ to 30 mmHg, further increasing the isoflurane concentration and inhaled nitric oxide (NO). With these interventions mPAP fell to 40 and CVP to 31 however the TR remained severe; CI in fact improved to 3.5–4.5 at this time. On discussion with the surgical team the decision was made to proceed with transplantation with the knowledge that without transplantation the patient’s likelihood of survival was extremely low, and that the TOE findings of RV dilatation, raised LAP (LA end systolic diameter 4.5 cm [normal < 3.3 cm]; interatrial septum fixed to the right [7]), and absence of left heart disease, suggested a hypervolemic state as a significant but reversible contributor to the degree of TR.

**Fig. 2:**
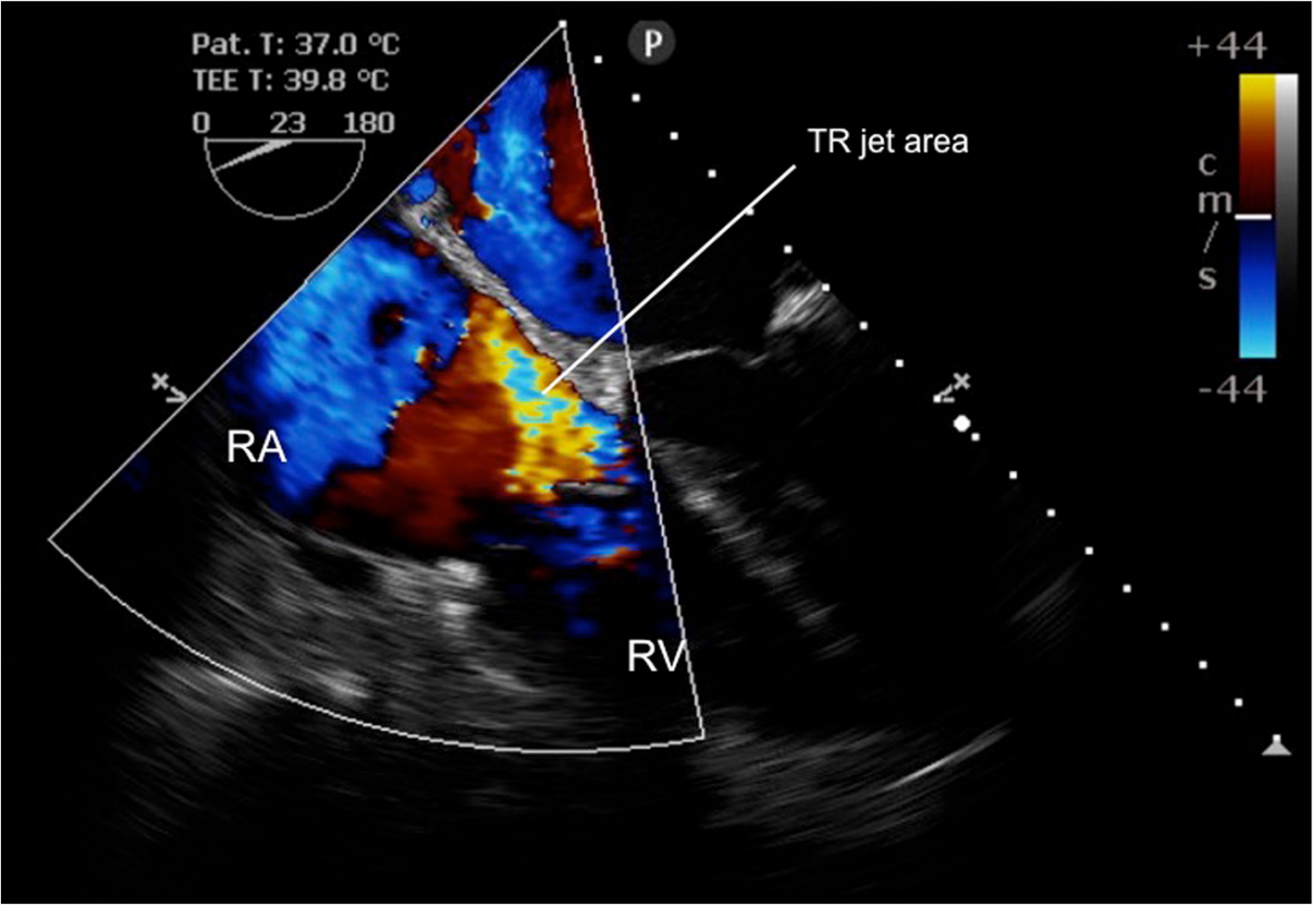
Mild tricuspid regurgitation, mid-oesophageal 4-chamber view at frame of maximal systolic TR jet area, achieved by anhepatic phase of OLT


Surgery proceeded with the understanding that significant blood loss would occur secondary to the elevated systemic venous pressures. Carefully monitored, permissive surgical hypovolemia was planned. As expected more than 9 l blood loss occurred within the first three hours which dramatically improved the TR severity to mild (jet area 4.3cm^2^; no vena contracta visible), with improvement of the RV dilatation (RV end diastolic area 40cm^2^), by the time of the anhepatic phase (Fig. 2, Additional file 2: Movie Clip 2). Concomitantly, mPAP fell to 25–30 mmHg and mean CVP to 15 mmHg. Inodilator therapy with intravenous milrinone had been prepared for further pulmonary vasodilation but was never required.

**Additional file 2**: Movie Clip 2 (MP4 11645 kb)


The remainder of the operation proceeded uneventfully with ongoing mild TR and normal RV size and systolic function. Blood loss continued to be an issue but was managed with judicious volume replacement whilst monitoring for worsening of TR and RV dilatation on TOE. Total infused volume was 23 l, consisting of 6.4 L of autologous red cells, 2.3 L of packed red blood cells, 10 L of Plasmalyte-148 (Baxter International Inc., Deerfield IL), 1.7 L of 20% albumin, 5 units of fresh frozen plasma, 10 units of cryoprecipitate and 1 bag of pooled platelets.

The patient recovered well postoperatively with serum creatinine stabilising at 106 micromol/L and a formal post-operative TTE showing normal RV and LV size and systolic function, and no significant valvular dysfunction. The patient was discharged to rehabilitation on day 13 post-transplantation and was well at follow up 2 years later.

## Discussion and conclusions

**Fig. 3:**
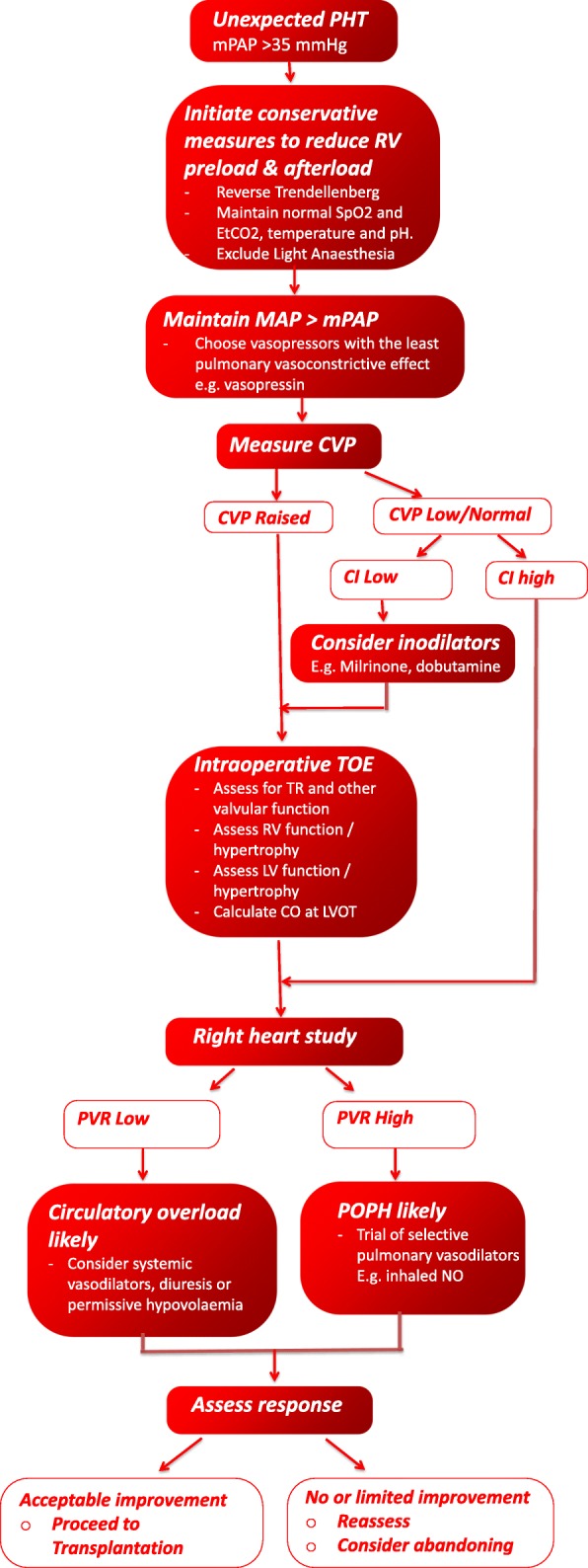
Recommended approach to unexpected severe TR and PHT on day of surgery. *CI is systematically underestimated by approximately 20% by PAC derived thermodilution in severe TR

We present a case of successful diagnosis, acute assessment and management of severe TR and undifferentiated PHT in a patient presenting for OLT with grossly elevated systemic venous pressures. Use of TOE was critical in diagnosing severe functional TR, RV volume overload, and establishing normal RV and LV systolic function and providing critical haemodynamic information on the response to interventions aimed at reducing the TR and PHT. This ultimately led to the correct decision to proceed with surgery despite a very high mortality risk. Figure 3 shows our approach to unexpected PHT at time of operation for OLT. It draws on previous work from Fukazawa [8] & Koh [9] and provides detail regarding the utility of TOE assessment and a framework for therapeutic options. One important advantage of TOE is that it can determine left-sided cardiac output using left ventricular outflow tract (LVOT) velocity-time integral calculations which are more accurate than PAC-derived right heart cardiac output (CO) measures when severe TR is present, which underestimates CO [10].

**Fig. 4:**
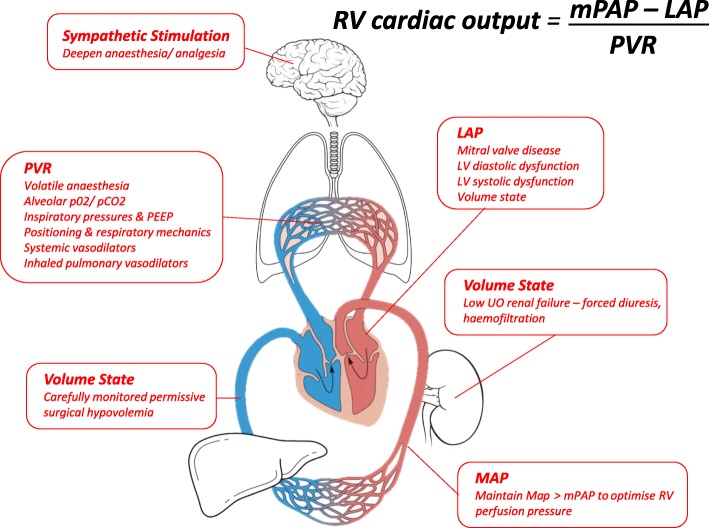
Multi-systems factors and interventions affecting severity of TR and PHT

Whilst the influence of PHT on perioperative risk has received much attention, less has been directed at the issue of co-existing TR and its compounding effects in the liver transplant population. Although the independent association of TR severity with liver function impairment has been established [1], some authors report increased post transplant mortality with pre-transplant TTE grading of TR of more than mild [11, 12]) whilst others did not [13]. Functional TR is worsened by increased right ventricular loading conditions, increased PVR and LAP being the most common causes of increased RV afterload. Once primary, or structural TR (defined as regurgitation due to compromise of tricuspid valve leaflet integrity) has been excluded by echocardiography, attention should be focused on reducing RV preload (ie. systemic venous return) and RV afterload (by reducing LAP and/ or PVR). In the perioperative period this would include consideration of inhaled or intravenous pulmonary vasodilators, or optimization of volume state by daily fluid restriction, diuretic therapy or haemofiltration. Table 1 summarises the role of intraoperative TOE in assessing severe unexpected TR and PHT during OLT. Figure 4 summarises the multi-system factors and interventions that influence severity of TR and PHT.

**Table 1:** Role of intraoperative transoesophageal echocardiography in assessment and management of severe tricuspid regurgitation during liver transplantation

Exclude structural TR	2D imaging	• Normal appearance and motion of tricuspid valve leaflets and para-annular structures
Exclude severe functional tricuspid regurgitation	Colour flow doppler	• Jet area > 10 cm^2^ • Large proximal flow convergence • Vena contracta width > 0.7 cm
	Spectral doppler	• Systolic flow reversal in hepatic veins • Dense TR signal with short deceleration time • Tricuspid inflow E wave > 1 cm/s • Effective Regurgitant Orifice Area > 0.4 cm^2^ • Regurgitant Volume > 45 ml
Assess RV systolic function	• RV fractional area change • TAPSE • RV systolic myocardial velocity	
Assess degree of RV and tricuspid annular dilatation	• RV End Diastolic Area • RV End Diastolic diameter (basal, mid, apical) • Tricuspid Annulus Diameter	
Assist quantification of left heart disease driving PHT	• Estimate LAP (LA size, inter-atrial septum mobility, E/e’) • Assess LV diastolic and systolic function • Quantify severity of mitral regurgitation/ stenosis	
Monitor improvement in the above indices in response to interventions reducing:	• RV preload (eg. systemic venous blood volume; positioning) • RV afterload (eg. pulmonary vascular blood volume; pulmonary vascular resistance; interventions to improve LAP)

The failure to diagnose this patient’s severe TR and PHT during her waitlist period reflects the rapidly progressive nature of these conditions, their self-perpetuating effects, and the complex pathophysiological interplay between CLD and TR/ PHT. Indeed, this was highlighted in Saragai’s study where there was a large discrepancy between estimated PAP by PAC at operation and historical TTE PAP measurement [14]. Additionally, clinical signs and sequelae of TR and PHT are non-specific and may be incorrectly attributed to worsening hepatic function. These sequelae include worsening LFTs, fluid overload, peripheral oedema and renal dysfunction. Had TTE been performed at the time of her initial deterioration, severe TR/ PHT could have been identified earlier and right heart catheterization would have been performed [15]. Attempts at optimizing volume state and pulmonary vascular resistance may have resulted in improved right heart haemodynamics by the time of surgery. The role of early right heart catheterization cannot be overemphasized as it provides the most accurate estimation PVR and the potential for determining reversibility of PHT over time, possibly influencing the decision for earlier transplantation. Although terlipressin has a strong evidence base for improving renal perfusion in cirrhotic liver disease by constricting splanchnic beds and mobilizing blood to the systemic circulation, undesirable effects on PHT have been demonstrated [16]. In retrospect, the deterioration in liver and kidney function in response to terlipressin as occurred twice in this case was indicative that the patient had TR and or significant volume overload and should have prompted further investigation.

With regards to the ideal timing of repeat TTE whilst on the waiting list, there is little evidence of the optimal timing and no priority given to specifically excluding TR/ PHT when apparent hepatic deterioration occurs. Although our institutional protocol suggests repeating a TTE every year, in our patient’s case, her remote place of residence contributed to her loss to follow up for repeat TTE. We propose that any patients waitlisted for OLT who are admitted with a decompensation of their CLD, or require terlipressin therapy, have TTE performed to screen for new or worsening TR/ PHT that may be a contributing and/ or compounding factor to hepatic decompensation.

When pulmonary hypertension is diagnosed in patients with cirrhosis, it is important to distinguish whether the cause is due to circulatory overload or from POPH, as the treatments and prognoses are different. Right heart catheterization with measurement of PVR and pulmonary capillary wedge pressure (PCWP) has been recommended [17], but this was not helpful in our case as we could not wedge the PAC. The inability to wedge a PAC has been reported to occur in up to 12% of cases [18], and in this case was most likely due to a hypervolemic pulmonary circulation causing dilatation of the pulmonary arterial tree to a wider dimension than that of the PAC balloon. Other causes of PHT, including pulmonary emboli, severe respiratory disease and left ventricular failure, should also be considered.

In patients awaiting OLT with significant PHT, a trial of systemic or pulmonary vasodilator therapy can help in the decision-making process to distinguish responders from non-responders [8, 17]. Inhaled NO alone did not provide reassurance in our case. However, increasing pulmonary vasodilation with increased isoflurane administration, reverse Trendellenberg positioning and carefully monitored permissive surgical hypovolemia caused the PHT and TR to improve (Fig. 4). The reversibility in PHT with these manoeuvres suggested that the cause of the PHT in our patient was circulatory overload, which confers a better prognosis. Koh and colleagues [9] achieved a similar result by emergently draining 9 l of ascites from the patient in their case report of acutely reversible PHT. Park and colleagues have shown that PHT that resolves during transplantation confers a prognosis similar to patients without PHT [19]. Other means of demonstrating reversibility by reducing venous return in the acute preoperative period could have also been achieved with a trial of diuretics or haemofiltration. The remarkable intra-operative resolution of the TR, from severe to mild, coupled with a concomitant reduction in RV size, suggests that the TR was at least partially attributable to RV dilatation.

In conclusion, we have demonstrated that use of intraoperative TOE in addition to other invasive monitors can successfully guide the acute diagnosis, on-table management and demonstration of reversibility of unexpected severe TR and PHT just prior to orthotopic liver transplantation. This assisted the ultimate decision to proceed with transplant and achieved successful transplantation with a good postoperative patient outcome. Nevertheless, every effort should be made to detect new onset severe TR and PHT in OLT waitlisted patients by performing regular TTE screening and having a high index of suspicion of TR and PHT at times of hepatic decompensation.

## Data Availability

Not applicable.

## Abbreviations

CI: Cardiac Index
CLD: Chronic Liver Disease
CO: Cardiac Output
CVP: Central venous pressure
LA: Left atrial
LAP: Left atrial pressure
LFTs: Liver function tests
LV: Left ventricle; Left ventricular
LVOT: Left Ventricular Outflow obstruction
MAC: Minimum alveolar concentration
MAP: Mean arterial pressure
MELD score: Model for End Stage Liver Disease score
mPAP: Mean pulmonary arterial pressure
MR: Mitral regurgitation
NASH: Non-alcoholic steato-hepatitis
NO: Nitric oxide
OLT: Orthotopic liver transplant
PAP: Pulmonary arterial pressure
PCWP: Pulmonary capillary wedge pressure
PHT: Pulmonary hypertension
POPH: Porto-pulmonary hypertension
PVR: Pulmonary vascular resistance
RV: Right ventricle; right ventricular
sPAP: Systolic pulmonary arterial pressure
TAPSE: Tricuspid annular plane systolic excursion
TOE: Trans-oesophageal echocardiography
TR: Tricuspid regurgitation
TTE: Trans-thoracic echocardiography
